# Prolonged insulin-induced hypoglycaemia reduces ß-cell activity rather than number in pancreatic islets in non-diabetic rats

**DOI:** 10.1038/s41598-022-18398-z

**Published:** 2022-08-18

**Authors:** Vivi F. H. Jensen, Anne-Marie Mølck, Jette Nowak, Johannes J. Fels, Jens Lykkesfeldt, Ingrid B. Bøgh

**Affiliations:** 1 Section for Experimental Animal Models, Department of Veterinary and Animal Sciences, University of Copenhagen, Copenhagen, Denmark; 2 Department of Safety Sciences & Imaging, Toxicology Development Projects, Novo Nordisk A/S, Novo Nordisk Park, 2760 Måløv, Denmark; 3 Department of Research Bioanalysis, Novo Nordisk A/S, Maaloev, Denmark

**Keywords:** Metabolism, Endocrine system and metabolic diseases, Cell biology, Endocrinology

## Abstract

Pancreatic β-cells have an extraordinary ability to adapt to acute fluctuations in glucose levels by rapid changing insulin production to meet metabolic needs. Although acute changes have been characterised, effects of prolonged metabolic stress on β-cell dynamics are still unclear. Here, the aim was to investigate pancreatic β-cell dynamics and function during and after prolonged hypoglycaemia. Hypoglycaemia was induced in male and female rats by infusion of human insulin for 8 weeks, followed by a 4-week infusion-free recovery period. Animals were euthanized after 4 or 8 weeks of infusion, and either 2 days and 4 weeks after infusion-stop. Total volumes of pancreatic islets and β-cell nuclei, islet insulin and glucagon content, and plasma c-peptide levels were quantified. Prolonged hypoglycaemia reduced c-peptide levels, islet volume and almost depleted islet insulin. Relative β-cell nuclei: total pancreas volume decreased, while being unchanged relative to islet volume. Glucagon: total pancreas volume decreased during hypoglycaemia, whereas glucagon: islet volume increased. Within two days after infusion-stop, plasma glucose and c-peptide levels normalised and all remaining parameters were fully reversed after 4 weeks. In conclusion, our findings indicate that prolonged hypoglycaemia inactivates β-cells, which can rapidly be reactivated when needed, demonstrating the high plasticity of β-cells even following prolonged suppression.

## Introduction

Blood glucose is the main factor controlling pancreatic insulin secretion and β-cell function, furthermore, changes to blood glucose levels appear to influence the maintenance of β-cell mass^1^. Postnatally, β-cell proliferation is thought to decline significantly after infancy in humans, mice and rats^2–8^; however, β-cells seem to have an extraordinary ability to rapidly adapt to food- and activity-related changes in circulating glucose levels by changing insulin secretion depending on metabolic needs^9^. These rapid changes to circulating insulin levels in response to alterations in blood glucose levels have been well characterised; however, the effects of prolonged metabolic stress on β-cell dynamics are still not completely understood. Currently, focus is increasing on the complex functional dynamics of β-cells in response to metabolic stress, as well as the potential return to normal function when the metabolic stress has resolved, to understand e.g. the β-cell failure seen in Type 2 Diabetes and restoration of function following weight loss^10^.

In mice and rats, chronic hyperglycaemia can cause β-cell hyperplasia and hypertrophy, while chronic hypoglycaemia may lead to pronounced β-cell hypotrophy and/or β-cell death^11–15^, indicating high plasticity of β-cell mass. This is further illustrated by the rapid transition from reductions in insulin secretion during acute hypoglycaemia to an increase following a return of glucose supply. It has been shown experimentally that in rats fasted for 72 h, β-cells had decreased intracellular insulin stores and synthesis capacity in response to glucose administration, an effect reversed within only 6 h of refeeding^16^. Others have described pancreatic islet atrophy and decreased β-cell size and function in insulin-infused hypoglycaemic mice after 10 days, where β-cell function and mass returned to normal within a week after insulin withdrawal^12^. However, the effects of long-term metabolic stress, such as continuous and prolonged insulin-induced hypoglycaemia for several weeks/months, on β-cell function and mass has to our knowledge not been investigated so far, neither has the potential ability to restore normal function following a prolonged state of continuous hypoglycaemia.

We have previously established a rat model of continuous insulin-induced hypoglycaemia using insulin-infusion, that enables the study of plasticity of β-cell mass and insulin production during prolonged metabolic perturbation of glucose metabolism as well as reversibility of changes^17^. Using this model, we showed that eight weeks of continuous hypoglycaemia was accompanied by microscopically recognizable atrophy of the pancreatic islets of Langerhans, which was reversible within four weeks after ending insulin-infusion^17^.

The purpose of the present study was to investigate this atrophy further and expand our understanding of the regulation of pancreatic β-cell dynamics and insulin-producing function by continuous prolonged suppression of blood glucose levels as well as reversal to normoglycaemia. We therefore performed the present study, where we investigated changes in β-cell mass and insulin production during and after this state. Furthermore, we included additional time-points during and after infusion to evaluate progression of changes during insulin-infusion and reversibility following insulin withdrawal. Eight weeks continuous infusion of insulin was chosen to ensure islet atrophy, as shown previously. An evaluation after four weeks was included to follow the development; and has been shown to reduce β-cell mass in mice following insulin-infusion via subcutaneous insulin pellets has shown to reduce β-cell mass in mice^18^. Following cessation of insulin-infusion in our model, transient rebound hyperglycaemia has been observed, with normalisation of blood glucose level within few days^17^. A similar finding is seen in rats following removal of insulinomas, with decline to normal levels in 2–4 days^19^ and following removal of subcutaneous insulin pellets in mice after ten days, where normalization was seen within a week^12^. This suggests a rapid return to function by the β-cells, we therefore sought to evaluate what happens to β-cell mass and insulin production as early as two days after ending insulin-infusion. To our knowledge, this is the first study to evaluate effects to β-cell mass following such an extended period of insulin-induced hypoglycaemia using a well-controlled, standardized, and reproducible model with individual adjustment of doses, as opposed to insulin implants, and without any confounding factors potentially accompanying insulinomas; the hypoglycaemia was consistent throughout the eight weeks. Also novel is the different time-points included, allowing assessment for short- and long-term recovery. Furthermore, both males and females were included to evaluate for sex differences in the response.

We hypothesized that: (i) prolonged insulin-induced hypoglycaemia causes a compensatory decrease in β-cell insulin production and secretion accompanied by decreased overall mass of β-cells and pancreatic islets, (ii) following withdrawal of exogenous insulin, normal β-cell mass as well as insulin production and secretion will be re-established, and (iii) islet glucagon content will increase during hypoglycaemia and normalize after end of dosing.

## Materials and methods

### Study design

**Figure 1:**
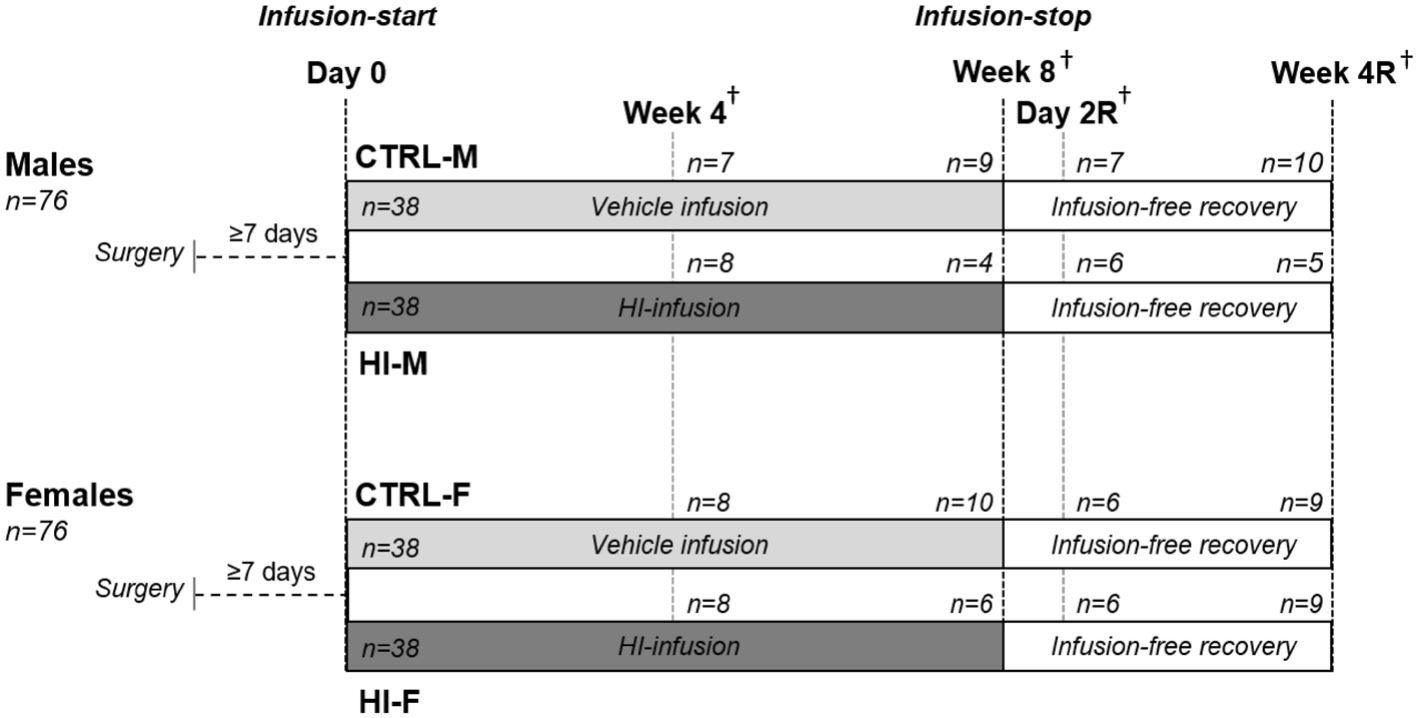
Study design. The study included male and female rats infused (surgically implanted intravenous catheter) with either vehicle or HI, resulting in a total of four groups: CTRL-M, HI-M, CTRL-F, and HI-F. Grey areas indicate the infusion period. Dark grey areas: Infusion with HI, light grey areas: Vehicle infusion. Infusion ended after 8 and was followed by an infusion-free recovery period. During infusion, animals were euthanized after 4 and 8 weeks. Following infusion-stop, animals were euthanized after 2 Days (Day 2R) or 4 weeks (Week 4R). † indicate euthanasia of animals. HI, human insulin; R, recovery.

The study design is depicted in Fig. 1 and Supplementary Table S1 and has been described in detail elsewhere as the pancreatic tissue used for the present investigations was sampled from animals of a previous study^20^. Briefly, 7–8 week-old male and female (n = 76/sex) Sprague Dawley rats (Crl:CD (SD), Charles River Limited, UK) were surgically implanted with a venous catheter, as described previously^21^. After at least seven days of post-surgery recovery, at approximately 8–9 weeks of age, intravenous infusion with either vehicle (control, CTRL) or human insulin (HI) (supplied by Novo Nordisk A/S, Maaloev, Denmark) was started (designated Day 1). Infusion lasted for 4 or 8 weeks before euthanasia (n-values stated in Fig. 1). Additionally, some animals received infusion for 8 weeks followed by an either 2-day or 4-week infusion-free recovery period (designated Day 2R and Week 4R) before euthanasia. Each time-point included four groups, vehicle- or HI-infused males (CTRL-M, HI-M) and females (CTRL-F, HI-F). Initial doses of HI were 72 and 54 nmol/kg/day in males and females, respectively, to compensate for differences in sensitivity to hypoglycaemia with a dose reduction to 60 and 48 nmol/kg/day after 12–19 days of infusion due to clinical signs of severe hypoglycaemia and premature deaths attributed to hypoglycaemia in some of the animals. Doses were higher in males as they have lower insulin sensitivity compared with females^21^. Animals were euthanized by carbon dioxide asphyxiation with subsequent exsanguination. After euthanasia, the pancreas was removed, weighed, and fixated in 10% neutral buffered formalin until further processing (see below).

The study was approved by ethics committee of Animal Welfare & Ethical Review Body of Envigo UK. All experimental procedures involving live animals have been ethically approved by the Animals (Scientific Procedures) Act 1986 (ASPA) and United Kingdom Secretary of State and were performed according to the ARRIVE guidelines, Directive 2010/63/EU, EC Commission Directive 2004/10/EC of the European Parliament, OECD Principles and Good Laboratory Practice 1998, and The Good Laboratory Practice (Codification Amendments Etc.) Regulations 2004 SI 2004/944, as well as Envigo (now Labcorp) and Novo Nordisk A/S company policies on the care and use of laboratory animals.

### Blood glucose level

Glucose was quantified in plasma and whole blood, as described previously^20^.

#### Plasma levels

Blood was sampled from the sublingual vein at regular intervals (at 0.25, 0.5, 6, 12, and 24 h, relating to start/end of infusion) following infusion-start on Day 1 and following infusion-stop at Week 8, i.e. during the first day of recovery, Day 1R (1–2 animals/time-point), to monitor for decreases and increases in blood glucose levels after infusion-start and -stop, respectively. Levels were quantified using the hexokinase method^21^.

#### Whole blood levels

To monitor blood glucose levels throughout the study, whole blood glucose levels were measured (tail vein) in all animals twice weekly during the infusion-period and weekly during the infusion-free recovery period. A snap blood glucose-monitoring device, using a single drop of whole blood, was used^21^.

### Plasma C-peptide levels

Blood was collected at euthanasia of all animals from the sublingual vein (K3-EDTA as anticoagulant), the separated plasma was stored frozen (approximately − 20 °C) until analysis. Quantification of plasma c-peptide levels was performed using a luminescence oxygen channeling immunoassay developed internally at Novo Nordisk A/S, as previously described^22^. The LLOQ of the assay is 18 pmol/l. The results are given as a mean of two duplicate measurements. Values measured as < LLOQ are reported as 18 pmol/l.

### Tissue processing for immunohistochemistry and stereology

In summary (details included below), eight sections were evaluated per pancreas, sampled throughout the pancreas 2300 µm apart to allow for the entire pancreas to be represented. For each of these sections, 40% of the tissue area was evaluated (100% for total pancreas volume). A method modified from Paulsen et al.^23^ was used for further processing of the pancreas tissue. Formalin-fixed pancreases were carefully dissected free of any remaining adipose tissue. Hereafter, pancreas was placed in a biopsy bag (catalog no. 4223, Tissue-Tek® Biopsy Bags, small, Sakura Finetek Europe B.V., AJ Alphen aan den Rijn, The Netherlands) and carefully pushed down in the bottom of the bag, which was then rolled tightly around the tissue, creating a cylinder shape, allowing for unbiased random sampling of tissue throughout the entire pancreas. Hereafter, it was placed in a tissue cassette and dehydrated overnight. On the following day, the tissue “cylinder” was taken out of the bag cut into eight slices using a multi-knife (distance of 2300 µm between each blade). Hereafter each of these eight slices were placed sequentially on their cut surfaces in one single tissue cassette and embedded in paraffin (Supplementary Fig. S1). The top-section from each block was cut into two serial 4 µm thick sections. One section was used for insulin/glucagon double-staining and the second for Insulin/Nkx6.1 double-staining, as described below. Pancreas sections from one CTRL-M and one CTRL-F group animal sacrificed 4 weeks after infusion-start as well as three CTRL-F group animals sacrificed on Day 2R were not stained for insulin/glucagon by mistake. Thus, data from these animals are missing.

### Immunohistochemistry

Sections of pancreatic tissue were double-stained for insulin and glucagon or for insulin and Nkx6.1 (β-cell nuclei). Protocols are included in Supplementary material.

### Stereology

Stereology was performed using the Cavalieri principle^24^. Each of the eight pancreas tissue slices were used for volume estimates of total pancreas, pancreatic islets, insulin, glucagon, and β-cell nuclei. Stereology was performed using newCAST software (Visiopharm, Hoersholm, Denmark) by an observer blinded to group and animal identity. Settings for the counting are listed in Supplementary Table S2 and resulting mean coefficient of error for each parameter in Supplementary Table S3.

### Statistics

Each time-point was analysed separately. Absolute and relative pancreas weights, plasma c-peptide levels, and stereology results were analysed using a 2-way ANOVA (overall effects of HI, sex, and interaction). In case of statistically significant interaction or effect of any of the two factors, a post hoc Sidak’s multiple comparisons test was conducted. If data were not normally distributed (Shapiro–Wilk normality test), data were transformed (logarithmic or square root) and tested for normal distribution. For stereology, this comprised the following data: Relative insulin to islet volume, Week 4R (square root); relative insulin to β-cell nucleus volume, Week 4 (square-root), Week 4R (log-transformed); relative β-cell nucleus to islet volume, Week 4, Week 4R (log-transformed); relative glucagon to islet volume, Week 8, Week 4R (log-transformed). The datasets absolute insulin volume and relative insulin to islet volume on Week 4, were not normally distributed even after transformation and consequently, an unpaired two-tailed Mann Whitney test was used to compare the HI-infused groups with each of their control groups, males and females separately.

Plasma c-peptide levels for Week 4 and Week 8 were compared using an unpaired two-tailed Mann Whitney test as described above, as values too low to be detected (< LLOQ) were set at the nominal value of 18 pmol/l resulting in a truncated dataset that was not normally distributed.

A *p* value < 0.05 was considered statistically significant.

## Results

A total of 34/152 animals died prematurely (found dead or were sacrificed) during the study. Twelve of these deaths were related to hypoglycaemia, five due to poor clinical condition, 15 to issues with the infusion system, and two for unknown reasons (details described in^20^).

Food consumption increased in HI-infused animals during infusion in both sexes, with a corresponding increase in body weights (data not shown here,^20^). After infusion-stop at 8 weeks, food consumption decreased markedly below the control level with a gradual return to normal within 11 days. Body weights were normalised within four days after infusion-stop and were similar to controls for the remainder of the study^20^. Pancreas weights, absolute and relative to body weight, were not affected by HI-infusion; Females had higher relative pancreas weight compared to males (Supplementary Fig. S2).

### Blood glucose and HI levels

**Figure 2:**
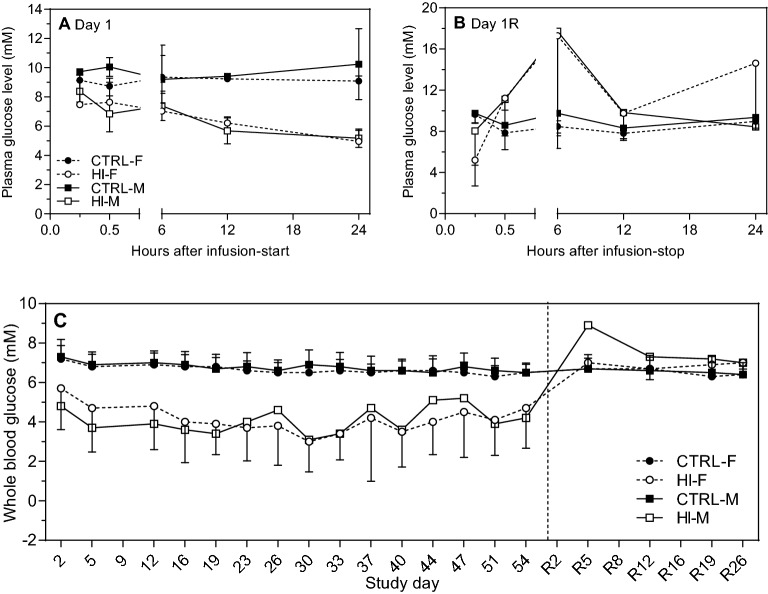
Blood glucose during and after infusion. Modified from^20^. (**A**) Plasma glucose levels after infusion-start on Day 1, n = 2/sex/time-point. (**B**) Plasma glucose levels after infusion-stop, i.e. Day 1 of recovery (Day 1R), n = 2/sex/time-point except in group HI-M at 12 h and 24 h: n = 1 (thus no SD for these time-points). (**C**) Whole blood glucose levels throughout the study, dashed line indicates infusion-stop. Day 2–23, n = 28–38/group; Day 26–51, n = 22–28/group; Day 54, n = 12–17/group; after infusion-stop (recovery period), n = 5–10/group. R, recovery.

*Plasma glucose levels* showed a nominal decrease within 6 h after infusion-start (20% in males, 25% in females, Fig. 2A). At 6 h following infusion-stop on Week 8, levels were increased above controls, but normalised within 12 h (Fig. 2B). *Whole blood glucose levels* were decreased throughout infusion. In females, levels normalised within five days following infusion-stop, but were higher than controls in males for at least five days (Fig. 2C); levels had normalised after 12 days. *Exposure to HI* was confirmed during the infusion period^20^.

### Plasma C-peptide levels

Plasma C-peptide levels were generally < LLOQ during HI-infusion in both males and females (Supplementary Fig. S3A + B), whereas levels had returned to control levels from two days after ending infusion (Supplementary Fig. S3C–D).

### Immunohistochemistry

**Figure 3:**
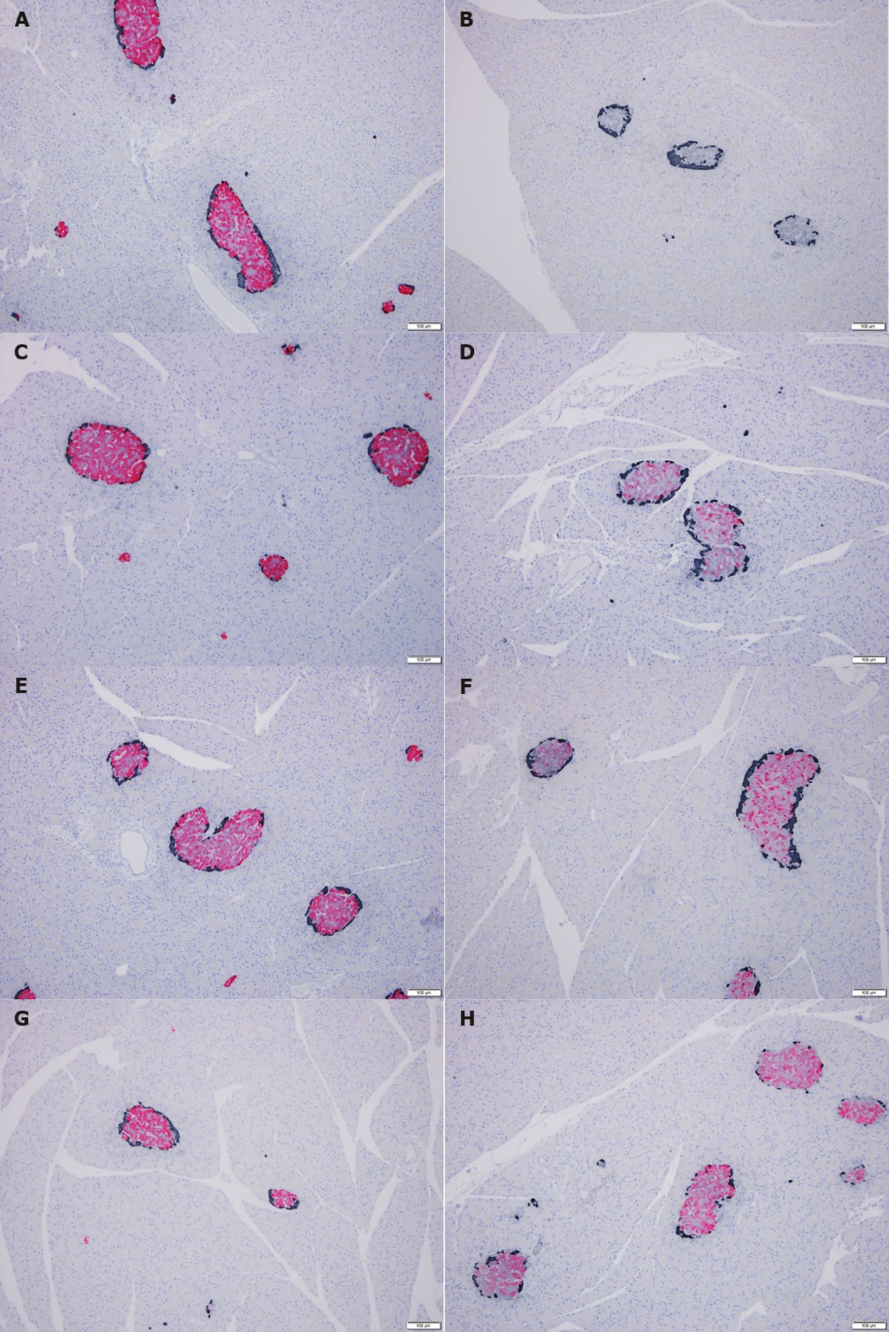
Microscopic images of pancreatic tissue double-stained for insulin (pink) and glucagon (black). Representative examples. Left panel: Controls, right panel: HI-infused animals. (**A**) + (**B**): Week 4, (**B**) is an example of islets completely negative for insulin staining. (**C**) + (**D**): Week 8, for some of the islets in the HI-infused animals insulin staining was completely absent. (**E**) + (**F**): Day 2R, (**G**) +(**H**): Week 4R. R, recovery (infusion-free period). Magnification: × 100, scalebar: 100 µm.

**Figure 4:**
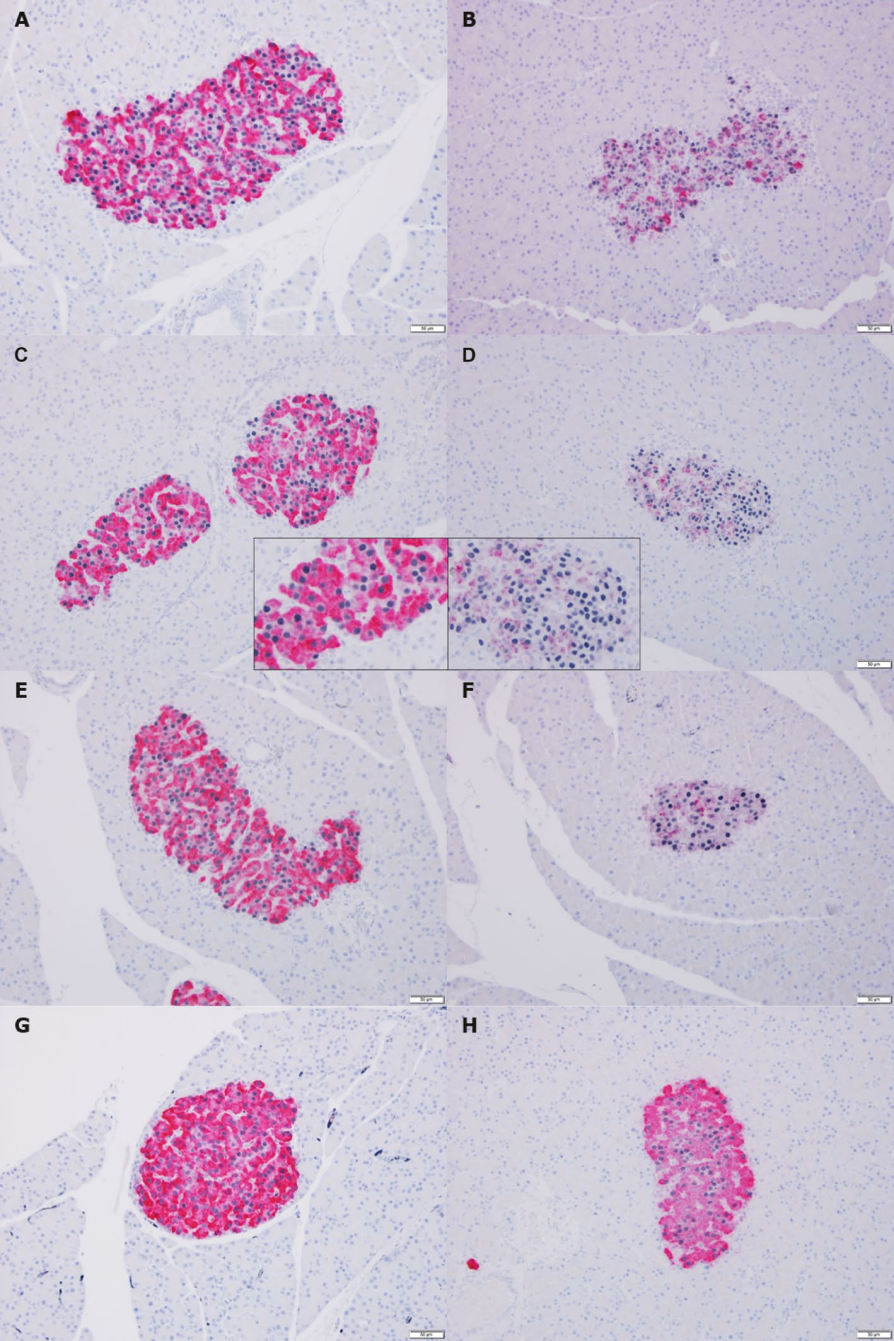
Microscopic images of pancreatic tissue double-stained for insulin (pink) and β-cell nuclei (black/dark purple). Representative examples. Left panel: Controls, right panel: HI-infused animals. (**A**) + (**B**): Week 4, (**B**) is an example of islets with occasional positive staining for insulin, islets completely negative for insulin staining were as in Fig. 3B. (**C**) + (**D**): Week 8. Insert: magnified portion of the same islet to illustrate distribution of nuclei, (**E**) + (**F**): Day 2R, (**G**) + (**H**): Week 4R. R, recovery (infusion-free period). Magnification: × 200, scalebar: 50 µm.

Representative images of insulin/glucagon and insulin/Nkx6.1 (β-cell nuclei*)* double-stained pancreatic tissue sections are depicted in Figs. 3 and 4, respectively. Insulin was generally absent or markedly decreased in pancreatic tissue sections from HI-infused animals, whereas glucagon-positive area seemed increased relative to islet size after infusion-start (Fig. 3A–B). Distribution of β-cell nuclei appeared more closely packed in HI-infused animals (Fig. 4A–B).

### Stereology

Absolute volumes of the parameters assessed are listed in Supplementary Table S4.

**Figure 5:**
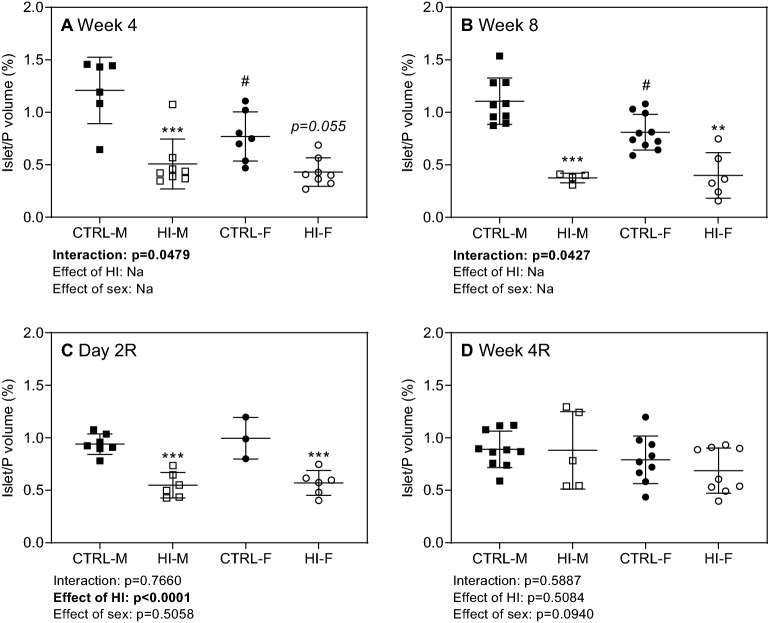
Relative islet to pancreas volume, individual (symbols) and means ± SDs. (**A**) Week 4: CTRL-M, n = 6; HI-M, n = 8; CTRL-F, n = 7; HI-F, n = 8. (**B**) Week 8: CTRL-M, n = 9; HI-M, n = 4; CTRL-F, n = 10; HI-F, n = 6. (**C**) Day 2R: CTRL-M, n = 7; HI-M, n = 6; CTRL-F, n = 3; HI-F, n = 6. (**D**) Week 4R: CTRL-M, n = 10; HI-M, n = 5; CTRL-F, n = 9; HI-F, n = 9. **p < 0.01, ***p < 0.001 versus control group for the same sex, #*p* < 0.05 versus CTRL-M. Abbreviations: HI, human insulin; Na, not applicable; P, pancreas; R, recovery (infusion-free period).

**Figure 6:**
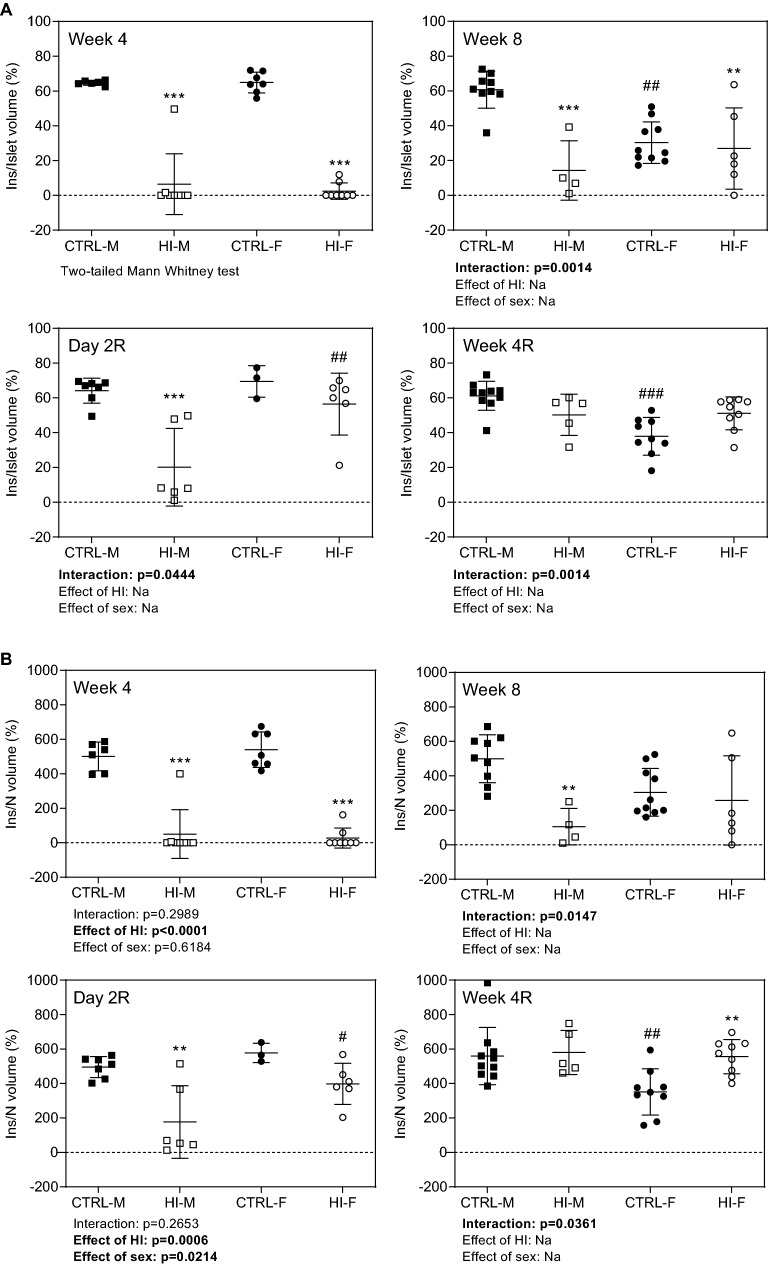
Relative insulin to islet and β-cell nucleus volume, individual (symbols) and means ± SDs. (**A**) Relative insulin to islet volume, (**B**) Relative insulin to β-cell nucleus volume. *Week 4*: CTRL-M, n = 6; HI-M, n = 8; CTRL-F, n = 7; HI-F, n = 8. *Week 8*: CTRL-M, n = 9; HI-M, n = 4; CTRL-F, n = 10; HI-F, n = 6. *Day 2R*: CTRL-M, n = 7; HI-M, n = 6; CTRL-F, n = 3; HI-F, n = 6. *Week 4R*: CTRL-M, n = 10; HI-M, n = 5; CTRL-F, n = 9; HI-F, n = 9. *p < 0.05, **p < 0.01, ***p < 0.001 versus control group for the same sex. #*p* < 0.05, ##*p* < 0.01 versus the corresponding group of the opposite sex. Abbreviations: HI, human insulin; Ins, insulin; N, nucleus; Na, not applicable; R, recovery (infusion-free period).

*Relative total islet to pancreas volume* was decreased during infusion, at Week 4 and 8 (Fig. 5A + B), an effect still seen two days after infusion-stop, followed by normalization of volumes four weeks after infusion-stop (Fig. 5C + D). Similar effects were seen for *relative insulin to islet volume* (Fig. 6A): overall, after four weeks of HI-infusion, the ratio displayed a pronounced decrease in both males and females; this was also seen after eight weeks in males, whereas females displayed only a modest decrease. Two days after infusion-stop, levels remained decreased in males, whereas four weeks of infusion-free recovery normalized levels. The same changes were observed for *relative insulin to β-cell nuclei volume* (Fig. 6B), with the exception of an increased ratio in females four weeks after infusion stop.

**Figure 7:**
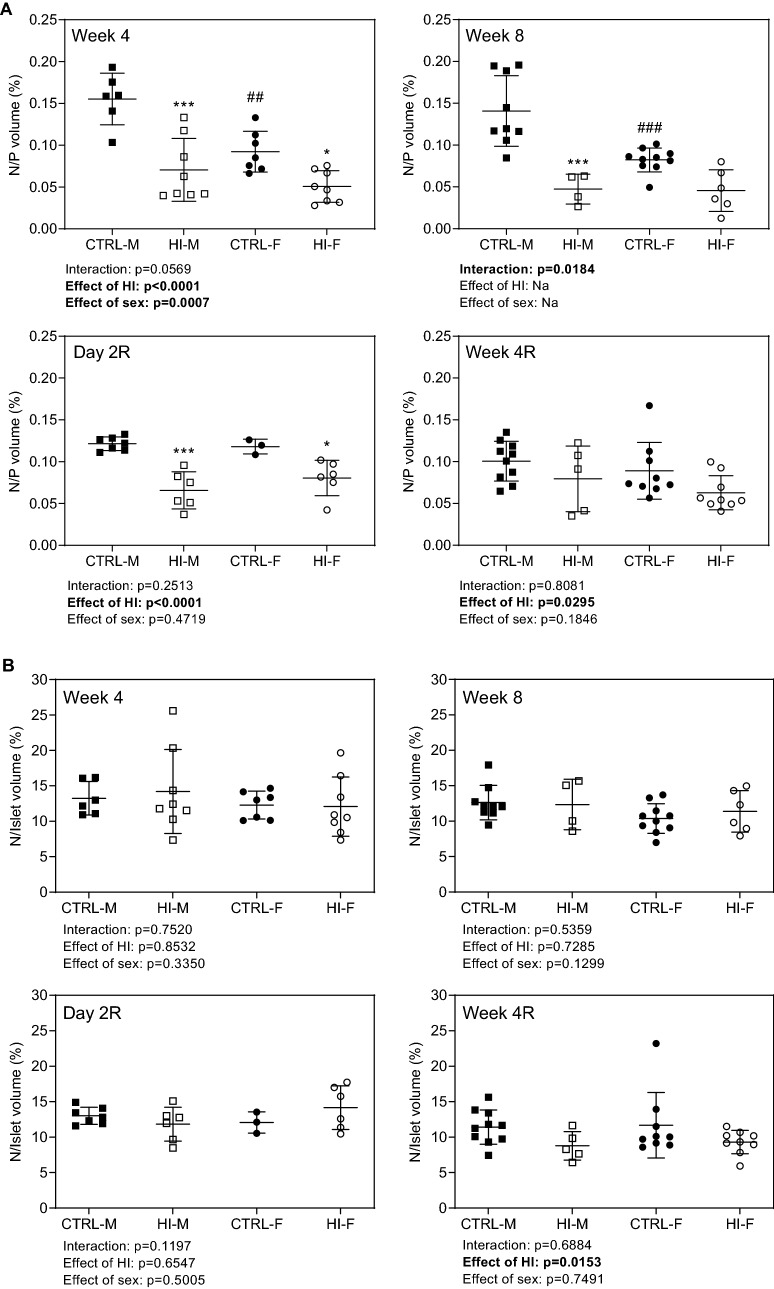
Relative β-cell nucleus to pancreas and islet volume, individual (symbols) and means ± SDs. (**A**) β-cell nucleus to pancreas volume, (**B**) β-cell nucleus to islet volume. *Week 4*: CTRL-M, n = 6; HI-M, n = 8; CTRL-F, n = 7; HI-F, n = 8. *Week 8*: CTRL-M, n = 9; HI-M, n = 4; CTRL-F, n = 10; HI-F, n = 6. *Day 2R*: CTRL-M, n = 7; HI-M, n = 6; CTRL-F, n = 3; HI-F, n = 6. *Week 4R*: CTRL-M, n = 10; HI-M, n = 5; CTRL-F, n = 9; HI-F, n = 9. *p < 0.05, ***p < 0.001 versus control group for the same sex. ##*p* < 0.01, ###*p* < 0.001 versus the corresponding group of the opposite sex. Abbreviations: HI, human insulin; N, nucleus; P, pancreas; R, recovery (infusion-free period).

*Relative β-cell nuclei to total pancreas volume* decreased during infusion (yet only modestly in females), still being decreased two days and four weeks after infusion-stop, though not significantly within each sex separately at Week 4R (Fig. 7A). *Relative β-cell nuclei to islet volume* was unaffected by insulin-infusion, with the only change being an overall decrease in previously HI-infused groups four weeks after infusion-stop (Fig. 7B).

**Figure 8:**
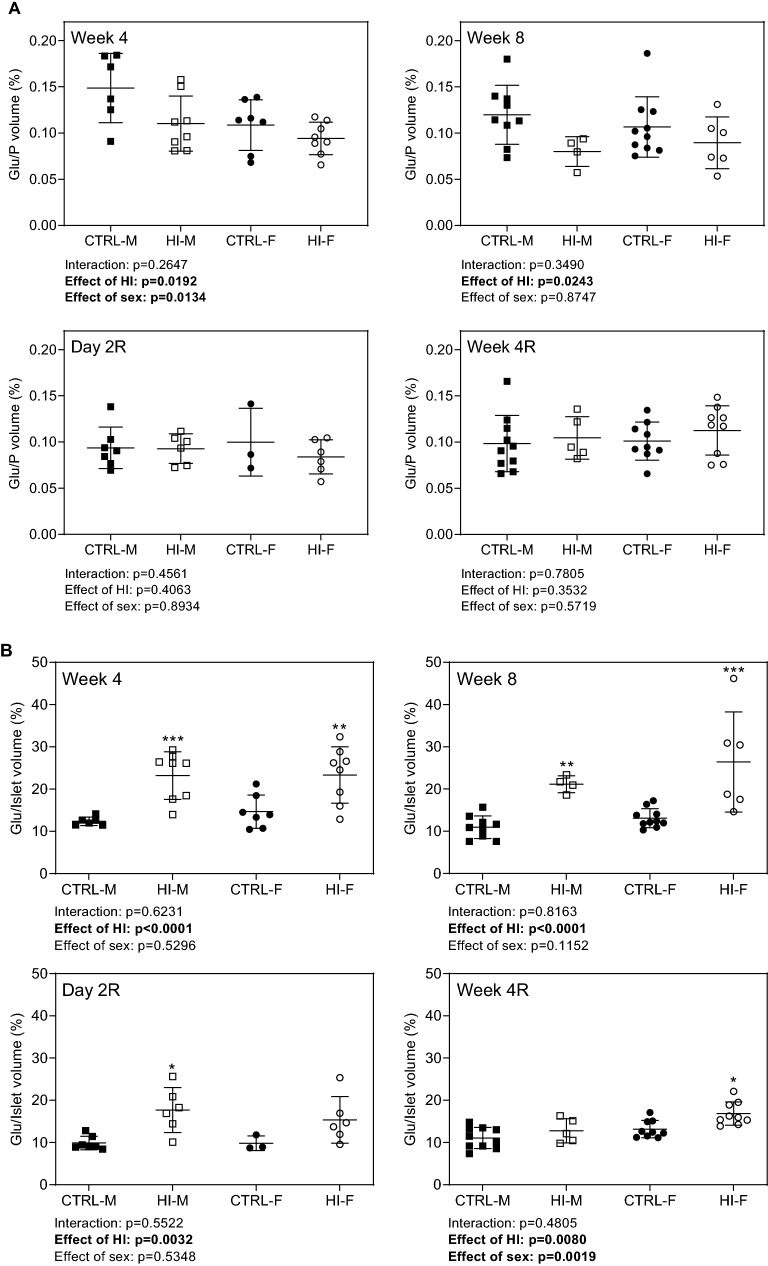
Relative glucagon to pancreas and islet volume, individual (symbols) and means ± SDs. (**A**) Glucagon to pancreas volume, (**B**) Glucagon to islet volume. *Week 4*: CTRL-M, n = 6; HI-M, n = 8; CTRL-F, n = 7; HI-F, n = 8. *Week 8*: CTRL-M, n = 9; HI-M, n = 4; CTRL-F, n = 10; HI-F, n = 6. *Day 2R*: CTRL-M, n = 7; HI-M, n = 6; CTRL-F, n = 3; HI-F, n = 6. *Week 4R*: CTRL-M, n = 10; HI-M, n = 5; CTRL-F, n = 9; HI-F, n = 9. *p < 0.05, **p < 0.01, ***p < 0.001 versus control group for the same sex. Abbreviations: Glu, glucagon; HI, human insulin; P, pancreas; R, recovery (infusion-free period).

HI-infusion decreased *relative glucagon to pancreas volume*, but not significantly within each sex separately, with no effects after infusion-stop (Fig. 8A); however, *relative glucagon to islet volume* increased during HI-infusion, an effect still seen two days after infusion-stop (Fig. 8B). At Week 4R, glucagon to islet volume had returned to normal in males, while still being increased in females. Generally, there was no statistically significant overall effect of sex on any of the stereologically evaluated parameters.

## Discussion

In the present study, we showed sustained β-cell adaption in response to prolonged continuous hypoglycaemia induced by insulin-infusion over eight weeks and a gradual return to normoglycaemia subsequent to infusion-stop. The results confirmed our hypotheses that hypoglycaemia is accompanied by adaptive decrease in β-cell insulin production, as reflected by decreased cytoplasmic β-cell insulin content and plasma c-peptide levels, as well as total β-cell mass. This effect was reversed within four weeks after end of infusion.

The β-cell insulin content was significantly decreased during insulin-infusion, recognized both by visual evaluation of pancreatic tissue sections stained for insulin, which displayed a very sporadic or even absent signal in islets, and by the quantitative decreased insulin volume relative to islet and β-cell nucleus volumes. Similarly, others have found absence of positive insulin-staining within islets following 10 days of continuous hypoglycaemia induced by subcutaneous insulin pellets in mice^12^, and that implanted insulinomas in rats are typically accompanied by a reduced amount of insulin^14,19,25^. The present findings likely reflect a decreased production causing depletion of cytoplasmic insulin, since hypoglycaemia is known to suppress endogenous insulin production through negative feedback^26,27^, and as supported by the accompanying low plasma c-peptide levels. Likewise, reduced levels of cytoplasmic insulin content in β-cells would explain the decrease in islet volume, also observed by qualitative histopathologic evaluation in our previous study^17^, possibly through a reduced cytoplasmic volume. Of note, when looking at the changes to relative insulin to islet volume and insulin to nucleus volume following insulin-infusion over time, insulin production is less suppressed after eight as compared to four weeks, suggesting that there may be some spontaneous recovery of insulin production at eight weeks. c-peptide levels were generally < LLOQ in these animals at this time-point, however, it could suggest that insulin production is slowly recovering, but insulin is not secreted due to the negative feed-back of the hypoglycaemia.

Another causative factor to the reduced islet volume may be the observed decrease in total β-cell nucleus volume relative to total pancreas volume during insulin-infusion. This is supported by the unchanged relative β-cell nucleus to islet volume, reflecting that decreases in absolute β-cell nucleus and islet volumes were proportional (e.g. for group HI-M: 46% and 51% at Week 4 and 61% and 63% at Week 8, respectively). The reduced β-cell nucleus to total pancreas volume is presumably due to β-cell hypoplasia and/or hypotrophy, which cannot be differentiated in the present study, since cell numbers were not quantified. But, evaluating the tissue sections, it could seem as if there was a higher β-cell nucleus density, suggesting hypotrophy rather than hypoplasia. This is supported by findings by others showing that decreased islet size is accompanied by shrunken β-cells with crowding of β-cell nuclei, following 10 days of continuous insulin-induced hypoglycaemia in mice^12^. β-cell nucleus hypotrophy could be caused by the down-regulated endogenous insulin production through decreased transcriptional activity, as reduced pancreatic pro- and prepro-insulin mRNA and insulin levels accompanies 5, 6, 12, and 28 days of insulin-induced hypoglycaemia in rats^28–31^. Therefore, the reduced β-cell nucleus volume likely reflects that β-cells are inactive/dormant in a non- or low-insulin producing state, e.g. caused by the continuous hypoglycaemia, with the “pool” of β-cells being generally maintained, ready for rapid reactivation by increases in blood glucose levels. This is supported by the rapid recovery of insulin production (plasma c-peptide levels) following infusion-stop. Re-establishment of normal β-cell number, i.e. through increased replication, would likely require a longer lag-time. However, it may not be completely clear-cut and could be a combination of both an overall reduction in β-cell activity and number, as others have found decreased β-cell mass and islet size combined with an increase in apoptotic insulin-positive β-cells following 14 days of insulin-infusion (subcutaneous pellets) in mice^18^. Mice with sub-renal transplanted pseudo-islets from a pancreatic β-cell line leading to severe hypoglycaemia displayed decreased overall mass of insulin-positive cells in endogenous islets with coinciding β-cell hypotrophy and an increase in apoptotic insulin-positive cells^32^; attenuated immunoreactivity of insulin in islets and blood glucose levels was normalized within four days after removal of exogenous islets, however, while β-cell size had returned to normal after 21 days, overall insulin-positive cell mass was still decreased. This supports, that in these mice, a combination of β-cell hypotrophy (decreased activity) and hypoplasia was present with fast re-establishment of insulin production in inactive β-cell and a more time-consuming replenishment of loss in β-cell population.

The transient hyperglycaemia seen following infusion-stop was most likely attributed to the suppressed endogenous insulin production, leaving the animals temporarily hypoinsulinaemic following withdrawal of exogenous insulin. This response has been shown previously following end of chronic insulin administration in rats in our model and by others^17,29^, and is a known response following removal of insulinomas in rats^14,15,19,25^. In the present study, whole blood glucose levels quickly returned to normal and plasma glucose levels within 24 h, likely reflecting a fast re-establishment of insulin production, as was confirmed by the normalised plasma c-peptide levels seen after two days. Conservation of β-cell number, either partly or completely, is probably responsible for this fast re-establishment of insulin production. Though relative insulin and β-cell nucleus volumes were still decreased two days after infusion-stop, the normal plasma c-peptide levels indicate that this could represent a delay in restoration of cytoplasmic insulin stores while re-establishing secretion. Presumably, reactivation of insulin production in dormant/inactive β-cells may have some lag-time prior to returning completely to normal. This imbalance was resolved within four weeks after infusion-stop as reflected by relative insulin, nucleus, and islet volumes. In line with this, others have seen restoration of β-cell function (insulin production) before β-cell mass following 10 days of continuous insulin-induced hypoglycaemia in mice^12^; this was interpreted as functional plasticity arising more rapidly than anatomical plasticity.

In our previous study, islet size area assessed by qualitative histology showed close to complete recovery within four weeks after infusion-stop (atrophy was still present in 3/11 animals)^17^, whereas in the present study, relative and absolute islet volumes were completely restored. This could reflect that—in the present study—quantitative methods were used, assessing islet volumes and including all parts of pancreas eliminating regional differences, since the same design was used and blood glucose levels were similar between the two studies.

An increased pancreatic glucagon positive volume in islet α-cells was expected, as this is an important hormone in the counter-regulatory response to hypoglycaemia through stimulation of hepatic glucose production^2^. In line with this, others have observed a doubling of pro-glucagon mRNA signal density in pancreatic islets concurrently with the decrease in pro-insulin signal following 12 days of insulin-infusion in rats^28^. Nevertheless, although relative glucagon to islet volume was increased during insulin-infusion here, an overall decrease in relative glucagon to total pancreas volume accompanied insulin-infusion. This could potentially reflect an increased secretion of glucagon from the α-cells, leading to a partial depletion of glucagon in the cytoplasm if the synthesis is not able to keep up despite of up-regulated production signals (and mRNA levels). Alternatively, glucagon production could be reduced due to the hyperinsulinaemia, as insulin is known to suppress glucagon secretion^27^. A study has shown complete absence of glucagon in islets in mice infused with insulin by pellets for 10 days^12^ and insulinoma-transplanted rats display a significant reduction in glucagon in islets after 21 days^14^, which could support either of the explanations. Unfortunately, plasma glucagon levels were not measured here, so this cannot be differentiated.

Several of the measured parameters displayed an effect of sex at some of the time-points, such as lower relative islet to pancreas volume (and absolute islet mass) in females in Week 4 and 8. This is in line with findings by others who found a higher percentage of islet tissue in pancreas in male versus female rats^33^. It could be connected to differences in function, as glucose-stimulated insulin secretion per unit islet cell mass in perfused pancreases was higher in the female rats^33^; likewise, isolated pancreatic islets from elderly female versus male rats as well as from women versus men display higher glucose-stimulated insulin secretion^34,35^. Possibly, the seemingly more efficient β-cells (in terms of insulin secretion) in females means that a lower number of cells is needed. Regardless, overall changes in the different parameters caused by HI-infusion were similar in males and females. Consequently, these occasional sex differences in parameters in controls are considered of little significance in the response to the continuous hypoglycaemia induced here. Though, notably, insulin relative to islet volume were increased in females during infusion, whereas males showed a trend for a decrease, at Week 4R. However, since c-peptide levels were not affected by previous HI-infusion in either sex after four weeks of recovery, this is likely due to the slight non-significant increase and decrease in absolute insulin and islet volumes, respectively, in females rather than an actual effect. The higher relative pancreas weight in females compared to males at all time-points likely reflects the higher body-weight of the males^20^.

A limitation of the present study is that the β-cell number was not assessed, meaning that it cannot be differentiated if the decreased nucleus volumes are mainly due to hypotrophy or hypoplasia with increased cell replication following infusion-stop. However, strengths include that the model comprises reproducible, well-controlled, and prolonged stable continuous hypoglycaemia with doses regulated according to individual body weight, as well as a long follow-up period; secondly, it included both male and female rats allowing assessment of potential differences in effects between sexes. Thirdly, the study used assessment of volumes, rather than descriptive histologic evaluation, providing quantitative results; fourthly, sampled tissue sections included parts of the entire pancreas using an unbiased sampling method according to stereological principles, giving a reliable estimate of volumes. This is important, as differences in response of insulin expression is seen in the pancreatic tail versus head in response to hyperinsulinaemic hypoglycaemia induced by insulinomas in rats^28^. Furthermore, different time-points during infusion and following infusion-stop were included, allowing progression of changes and assessment for short- and long-term recovery.

In summary, we propose that the reduced, but reversible, total mass of β-cell nuclei and islet volume as well as β-cell insulin production, predominantly represent an inactivation of β-cells rather than a reduced cell number, as the main response to prolonged hypoglycaemia, allowing for rapid reactivation. The same overall response was similar between male and female rats. Relevant future investigations include assessment of β-cell number, as well as staining for apoptotic and replicating cells, to differentiate if the observed decreased total volume of β-cell nuclei is due to hypotrophy or a decrease in cell number and if recovery of normal β-cell mass following return to normoglycaemia is accompanied by replication of β-cells. Likely, it could be a combination of the above. To our knowledge, this is the first study to evaluate effects to β-cell mass following such an extended period of insulin-induced hypoglycaemia using a well-controlled and standardized model. Also novel is the different time-points included, allowing assessment for progression of changes during hypoglycaemia and of short- and long-term recovery. Furthermore, both males and females were included to evaluate for sex differences in the response.

In conclusion, the present findings indicate that functional and morphological plasticity of β-cells is highly dynamic even following prolonged suppression of activity and that changes to the available β-cell mass, whether representing active cell volume or number, are reversible.

## Supplementary Information

Supplementary Information.

## Data Availability

The datasets used and/or analysed during the current study available from the corresponding author on reasonable request.
