# Supplementary material for: Prolonged insulin-induced hypoglycaemia reduces ß-cell activity rather than number in pancreatic islets in non-diabetic rats
Source: Sci Rep. 2022 Aug 18;12:14113. doi: 10.1038/s41598-022-18398-z (PMC9388517; doi:10.1038/s41598-022-18398-z)
Supplement: Supplementary file 1 — Supplementary Information. [file 41598_2022_18398_MOESM1_ESM.docx]

**Prolonged insulin-induced hypoglycaemia reduces ß-cell activity rather than number in pancreatic islets in non-diabetic rats**

**Supplementary material**

**Supplementary Table S1 Study design**

Modified from [1].

| **Termination** | **Group** | ***n*** | **Final *n***^a)^ | **Infusion** |
| --- | --- | --- | --- | --- |
| Week 4 | CTRL-M | *9* | *7* | Vehicle |
|  | HI-M | *9* | *8* | HI |
|  | CTRL-F | *9* | *8* | Vehicle |
|  | HI-F | *9* | *8* | HI |
|  |  |  |  |  |
| Week 8 | CTRL-M | *10* | *9* | Vehicle |
|  | HI-M | *10* | *4* | HI |
|  | CTRL-F | *10* | *10* | Vehicle |
|  | HI-F | *10* | *6* | HI |
|  |  |  |  |  |
| Day 2R | CTRL-M | *9* | *7* | - |
|  | HI-M | *9* | *6* | - |
|  | CTRL-F | *9* | *6* | - |
|  | HI-F | *9* | *6* | - |
|  |  |  |  |  |
| Week 4R | CTRL-M | *10* | *10* | - |
|  | HI-M | *10* | *5* | - |
|  | CTRL-F | *10* | *9* | - |
|  | HI-F | *10* | *9* | - |

^a)^ Number of animals at scheduled sacrifice, excluding premature dead animals. Day 2R and Week 4R, two days and four weeks of recovery (R) after infusion-stop.

**Supplementary Table S2** **Settings for stereologic counting**

| **Total**  **volumes** | **Immunohistochemical**  **staining** | **Magnification** |  | **Grid setting in software** | |  | **Sampling of total pancreas**  **area in tissue section** | **Shape**  **factor** |
| --- | --- | --- | --- | --- | --- | --- | --- | --- |
|  |  |  |  | **Groups** | **Points** |  |  |  |
| **Pancreas** | Insulin/glucagon | X50 |  | 1x1 | 2x2^a)^ |  | 100% | 4 |
| **Islets** | Insulin/glucagon | X400 |  | 4x4^a)^ | 1x3 |  | 40% | 4 |
| **Insulin** | Insulin/glucagon | X400 |  | 4x4 | 1x3^a)^ |  | 40% | 4 |
| **Glucagon** | Insulin/glucagon | X400 |  | 3x3 | 4x3^a)^ |  | 40% | 4 |
| **β-cell nuclei** | Insulin/Nkx6.1 | X400 |  | 6x6 | 2x4^a)^ |  | 40% | 4 |

^a)^ Used for counting of positive hit-points.

**Supplementary Table S3 Mean coefficient of error (CE) for each of the parameters estimated for volumes**

|  | **Mean CE** | **SD** | **Min** | **Max** |
| --- | --- | --- | --- | --- |
| **Pancreas** | 1.9% | 0.24% | 1.4% | 2.8% |
| **Islets** | 3.0% | 0.86% | 1.5% | 6.0% |
| **Insulin** | 3.4% | 2.2% | 1.2% | 11.7% |
| **Glucagon** | 3.0% | 0.60% | 1.7% | 4.5% |
| **β-cell nuclei** | 2.4% | 0.69% | 1.2% | 4.9% |

**Supplementary Table S4 Absolute volumes of each of the parameters assessed by stereology**

|  | **Mean±SD (mm^3^)** | | | |  |  | **2-way ANOVA** | | |
| --- | --- | --- | --- | --- | --- | --- | --- | --- | --- |
|  | **CTRL-M** | **HI-M** | **CTRL-F** | **HI-F** | *n* |  | **INT** | **HI** | **Sex** |
| **Pancreas** | |  |  |  |  |  |  |  |  |
| *Week 4* | 510.0±54 | 578.0±119 | 543.1±42 | 525.2±70 | *6-8* |  | NS | NS | NS |
| *Week 8* | 580.7±167 | 662.6±142 | 531.3±44 | 579.1±107 | *4-10* |  | NS | NS | NS |
| *Day 2R* | 701.2±102 | 637.4±85 | 595.4±169 | 560.8±100 | *3-7* |  | NS | NS | NS |
| *Week 4R* | 668.7±122 | 609.0±101 | 522.9±84# | 520.1±100 | *5-10* |  | NS | NS | *0.0038* |
| **Islets** | |  |  |  |  |  |  |  |  |
| *Week 4* | 6.08±1.5 | 3.01±1.9** | 4.23±1.6 | 2.25±0.8^a)^ | *6-8* |  | NS | *0.0001* | *0.0263* |
| *Week 8* | 6.27±1.6 | 2.44±0.2*** | 4.29±0.9## | 2.17±0.9** | *4-10* |  | NS | *<0.0001* | *0.0218* |
| *Day 2R* | 6.53±0.5 | 3.56±1.2*** | 5.82±1.6 | 3.18±0.8** | *3-7* |  | NS | *<0.0001* | NS |
| *Week 4R* | 6.00±1.7 | 5.16±1.6 | 4.02±0.9## | 3.43±0.7 | *5-10* |  | NS | NS | *0.0003* |
| **Insulin** | |  |  |  |  |  |  |  |  |
| *Week 4* | 3.93±0.9 | 0.47±1.3** | 2.73±1.0 | 0.09±0.2*** | *6-8* |  | NA (Mann Whitney test) | | |
| *Week 8* | 3.88±1.4 | 0.33±0.4*** | 1.34±0.7### | 0.66±0.6 | *4-10* |  | *0.0007* | NA | NA |
| *Day 2R* | 4.18±0.5 | 0.81±1.0*** | 4.11±1.5 | 1.85±0.8** | *3-7* |  | NS | *<0.0001* | NS |
| *Week 4R* | 3.71±1.3 | 2.62±1.0 | 1.59±0.7### | 1.72±0.3 | *5-10* |  | NS^b)^ | NS | *<0.0001* |
| **β-cell nuclei** | |  |  |  |  |  |  |  |  |
| *Week 4* | 0.760±0.14 | 0.408±0.24** | 0.501±0.15# | 0.261±0.09* | *7-8* |  | NS | *<0.0001* | *0.0023* |
| *Week 8* | 0.793±0.27 | 0.297±0.07*** | 0.438±0.09## | 0.255±0.14 | *4-10* |  | *0.0349* | NA | NA |
| *Day 2R* | 0.849±0.10 | 0.425±0.18*** | 0.618±0.21 | 0.444±0.11 | *6-7* |  | NS^c)^ | *<0.0001* | NS |
| *Week 4R* | 0.676±0.22 | 0.469±0.22 | 0.450±0.11# | 0.315±0.07 | *5-10* |  | NS | *0.0062* | *0.0027* |
| **Glucagon** | |  |  |  |  |  |  |  |  |
| *Week 4* | 0.748±0.20 | 0.633±0.20 | 0.593±0.17 | 0.170±0.10 | *6-8* |  | NS | NS | *0.0215* |
| *Week 8* | 0.664±0.17 | 0.513±0.02 | 0.563±0.17 | 0.170±0.12 | *4-10* |  | NS | NS | NS |
| *Day 2R* | 0.644±0.11 | 0.593±0.14 | 0.565±0.13 | 0.460±0.08 | *3-7* |  | NS | NS | *0.0483* |
| *Week 4R* | 0.644±0.17 | 0.623±0.09 | 0.519±0.09 | 0.565±0.08 | *5-10* |  | NS | NS | *0.0419* |

^a)^ p=0.0689 versus CTRL-F group. ^b)^ p=0.0594. ^c)^ p=0.0560 *p<0.05, **p<0.01, ***p<0.001 versus control group for the same sex. #p<0.05, ##p<0.01, ###p<0.001 versus the corresponding group of the opposite sex. INT, interaction. NA, not applicable. NS, no significant effect. R, recovery (infusion-free period).


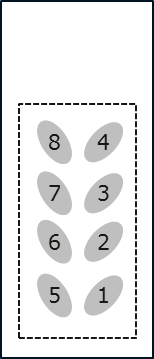


**Supplementary Figure S1 Order of pancreatic tissue sections on object glasses**

In order to be able to identify the sequence of pancreas sections (8/pancreas) for the stereologic evaluation, they were placed sequentially in the same order for each pancreas. Each of the grey areas represent a pancreatic tissue section, and the numbers represent the order of slices i.e. no. 1 is the first slide from each pancreas etc.

**Supplementary Figure S2 Pancreas weight relative to body weight,** **individual (symbols) and means±SDs**

**A:** Week 4, n-values: CTRL-M, n=7; HI-M, n=8; CTRL-F, n=8; HI-F, n=8. **B:** Week 8, n-values: CTRL-M, n=8; HI-M, n=3; CTRL-F, n=10; HI-F, n=4. Body weight was not recorded for one CTRL-M, one HI-M, and two HI-F group animals by mistake. **C:** Day 2R, n-values: CTRL-M, n=7; HI-M, n=6; CTRL-F, n=6; HI-F, n=6. **D:** Week 4R. n-values: CTRL-M, n=10; HI-M, n=5; CTRL-F, n=9; HI-F, n=9. #p<0.05, ##p<0.01 versus the corresponding group of the opposite sex.

**Supplementary Figure S3 Plasma C-peptide levels, individual (symbols) and means±SDs**

**A:** Week 4: CTRL-M, n=6; HI-M, n=8; CTRL-F, n=8; HI-F, n=8. The result from one CTRL-M group animal was excluded due to a high coefficient of variation for the duplicate measurements. **B:** Week 8: CTRL-M, n=9; HI-M, n=4; CTRL-F, n=10; HI-F, n=6. **C:** Day 2R: CTRL-M, n=7; HI-M, n=6; CTRL-F, n=5; HI-F, n=6. One animal in the CTRL-F group was not sampled by mistake. **D:** Week 4R: CTRL-M, n=10; HI-M, n=5; CTRL-F, n=9; HI-F, n=9. Levels measured as <LLOQ (18 pM) are reported as the nominal value of 18. **p<0.01 and ***p<0.001 versus control group for the same sex. Abbreviations: HI, human insulin; R, recovery (infusion-free period).

**Immunohistochemistry**

*Insulin/glucagon double-staining:* Sections of pancreatic tissue were double-stained for insulin and glucagon or for insulin and Nkx6.1 (β-cell nuclei). Dewaxed sections were heated in a microwave oven with TEG buffer (pH 9, AMPQ17020.5000, Ampliqon A/S, Odense, Denmark) for 15 min., followed by 15 min. of resting with buffer, and rinsed in demineralised water for 5 min. This was followed by incubation with a tris-buffered saline (TBS)/Tween 20 0.05% (TBST) solution (AMPQ40829.5000, Ampliqon A/S and P2287, Sigma-Aldrich Denmark Aps, Brøndby, Denmark) for 5 min. Following a 2 x5 min. rinse with TBST solution, endogenous peroxidase activity was blocked by incubation with Dako Peroxidase-Blocking Solution (S2003, Dako Denmark A/S, Glostrup, Denmark) for 10 min. Sections were rinsed 2 x 5 min. with TBST solution, pre-incubated with a TBST/1% BSA (A1662, Sigma-Aldrich Denmark Aps) solution for 30 min., which was then poured off, followed by 30 min. of incubation with a primary polyclonal guinea pig antibody against insulin (ab7842, abcam, Cambridge, UK), diluted in TBS+1% BSA to a concentration of 1:75. Following a rinse with TBST solution, sections were incubated with primary monoclonal rabbit antibody against glucagon (ab92517, abcam), diluted in TBS+1% BSA to a concentration of 1:6000. Hereafter, sections were rinsed in TBST for 2 x 3 min. and incubated for 30 min. with a secondary donkey anti-guinea pig antibody (706-055-148, Jackson ImmunoResearch Europe Ltd, Cambridgeshire, UK) diluted in TBS+1% BSA to a concentration of 1:500. After 2 x 3 min. of rinsing in TBST, sections were incubated for 30 min. with Vector Red AP Substrate (SK-5105, Vector Laboratories Ltd, Peterborough, UK) for 30 min. Following 2 x 3 min. of rinsing with TBST, sections were incubated for 30 min. with EnVision anti-rabbit HRP (K4011, Dako Denmark A/S), rinsed for 2 x 3 min. in TBST, and incubated for 15 min. with Vector SG Peroxidase (HRP) Substrate (SK-4700, Vector Laboratories Ltd). Sections were then rinsed for 2 x 3 min. with TBST, followed by rinsing in running tap water for 5 min. before applying a Mayers Haematoxylin counter-stain (30 sec.). After 5 min. of rinsing in running tap water, dried on a hot plate and then mounted in Pertex (41-4012-00, Medite GmbH, Burgdorf, Germany). Control of specificity of the antibodies consisted excluding either of the primary antibodies from the above steps to ensure that there was no overlap in the staining.

*Insulin/Nkx6.1 double-staining:* Sections of pancreatic tissue were double-stained for insulin and Nkx6.1 (β-cell nuclei). The same protocol as for insulin/glucagon double-staining was used, with the following exceptions: instead of primary antibody against glucagon, a primary monoclonal mouse antibody against Nkx6.1 (F55A12, Novo Nordisk A/S, Bagsværd, Denmark [13]) diluted to a concentration of 1:2000 was used, and EnVision anti-mouse HRP (K4007, Dako Denmark A/S) as the secondary antibody. Additionally, Mayers Haematoxylin counter-stain was applied for 15 instead of 30 sec.

**Stereology**

Settings for the stereologic counting for each parameter were based on preliminary counting on randomly selected sections, allowing for a minimum 150-200 hit-points in total for the eight tissue sections and a coefficient of error (CE) <10% for each parameter in each animal. Settings for each counting are listed in Suppl. Table 2. Volume and mean CE were calculated for each parameter according to stereologic principles (taking the systematic sampling and order of the tissue sections into account) using the newCAST software [14, 15]. For the insulin counts, CE for seven animals was >10% (range: 11.7-54.6%), distributed into HI-M and HI-F groups from Week 4 (n=2), Week 8 (n=2), and Day 2R (n=3), where the number of insulin hit-points ranged from 2-28 in total/pancreas. Therefore, pancreas slides from these animals were recounted using a denser grid to increase the number of hit-points (groups: 6x6, points 2x4, using points for counting), the remaining settings were as previously. After recount, the CE was <10% for four out of seven pancreases (4.6-9.0%, 137-194 hit-points/count), where the CE for the remaining three ranged between 10.5 and 11.7 % (19-25 hit-points/count). This small deviation was considered acceptable and without any impact on the outcome. The volume and CEs from these recounts substituted the results from the first counts in the statistical analysis. Overall, the mean CE for all parameters were between 1.9 and 3.4% (Suppl. Table 3), with no major differences between the mean CE for each group within each parameter.
